# The Role of the European Society of Human Genetics in Delivering Genomic Education

**DOI:** 10.3389/fgene.2021.693952

**Published:** 2021-09-03

**Authors:** Edward S. Tobias, Elena Avram, Patricia Calapod, Christophe Cordier, Johan T. den Dunnen, Can Ding, Vita Dolzan, Sofia Douzgou Houge, Sally Ann Lynch, James O’Byrne, Philippos Patsalis, Inga Prokopenko, Celia A. Soares, Adam P. Tobias, William G. Newman

**Affiliations:** ^1^Academic Unit of Medical Genetics and Clinical Pathology, Laboratory Medicine Building, Queen Elizabeth University Hospital, University of Glasgow, Glasgow, United Kingdom; ^2^School of Medicine, Dentistry and Nursing, College of Medical, Veterinary and Life Sciences, University of Glasgow, Glasgow, United Kingdom; ^3^Clinical Genetics, West of Scotland Centre for Genomic Medicine, Laboratory Medicine Bldg., NHS Greater Glasgow and Clyde, Queen Elizabeth University Hospital, Glasgow, United Kingdom; ^4^MedLife, Bucharest, Romania; ^5^Department of Medical Genetics, Faculty of Medicine, Lucian Blaga University of Sibiu, Sibiu, Romania; ^6^European Society of Human Genetics, Gothenburg, Sweden; ^7^Department of Genetics, CC-SYNLAB Suisse SA, Lausanne, Switzerland; ^8^Department of Human Genetics, Leiden University Medical Center, Leiden, Netherlands; ^9^Department of Clinical Genetics, Leiden University Medical Center, Leiden, Netherlands; ^10^Institute of Human Genetics, University Medical Centre of the Johannes Gutenberg University, Mainz, Germany; ^11^Pharmacogenetics Laboratory, Faculty of Medicine, Institute of Biochemistry and Molecular Genetics, University of Ljubljana, Ljubljana, Slovenia; ^12^Department of Medical Genetics, Haukeland University Hospital, Bergen, Norway; ^13^Manchester Centre for Genomic Medicine, Saint Mary’s Hospital, Manchester University NHS Foundation Trust, Manchester, United Kingdom; ^14^Division of Evolution and Genomic Sciences, School of Biological Sciences, University of Manchester, Manchester, United Kingdom; ^15^Clinical Genetics, Children’s Health Ireland at Crumlin and Temple Street, Dublin, Ireland; ^16^National Centre for Inherited Metabolic Diseases, Mater Misericordiae University Hospital, Dublin, Ireland; ^17^NIPD Genetics Limited, Nicosia, Cyprus; ^18^Department of Basic and Clinical Sciences, University of Nicosia Medical School, Nicosia, Cyprus; ^19^Department of Clinical and Experimental Medicine, University of Surrey, Guildford, United Kingdom; ^20^Department of Metabolism, Digestion and Reproduction, Imperial College London, London, United Kingdom; ^21^Centro de Genética Médica Jacinto Magalhães, Centro Hospitalar Universitário do Porto, Porto, Portugal; ^22^Unit for Multidisciplinary Research in Biomedicine, Instituto de Ciências Biomédicas Abel Salazar/Universidade do Porto, Porto, Portugal; ^23^Edinburgh Medical School, College of Medicine and Veterinary Medicine, University of Edinburgh, Edinburgh, United Kingdom

**Keywords:** education, genomics, European Society of Human Genetics, Education Committee, EuroGEMS, massive open online course, courses/diffusion, mentorship

## Abstract

The European Society of Human Genetics (ESHG) was founded in 1967 as a professional organisation for members working in genetics in clinical practice, research and education. The Society seeks the integration of scientific research and its implementation into clinical practice and the education of specialists and the public in all areas of medical and human genetics. The Society works to do this through many approaches, including educational sessions at the annual conference; training courses in general and specialist areas of genetics; an online resource of educational materials (EuroGEMS); and a mentorship scheme. The ESHG Education Committee is implementing new approaches to expand the reach of its educational activities and portfolio. With changes in technology, appreciation of the utility of genomics in healthcare and the public’s and patients’ increased awareness of the role of genomics, this review will summarise how the ESHG is adapting to deliver innovative educational activity.

## Introduction

The European Society of Human Genetics (ESHG^1^) was established in March, 1967, by a small group of European geneticists attending a conference in Chicago, United States (Renwick and Edwards, 1995). The first symposium of the new society was held in Copenhagen, Denmark, in November 1967, with invited lectures on genetic polymorphisms and a session for contributed papers. In 1993, the Society started to publish its own Journal, the European Journal of Human Genetics, which now appears monthly. The Society now has over 3,000 members, composed of Clinical Geneticists, Genetic Counsellors, diagnostic laboratory and research scientists, students and individuals from other disciplines with an interest in human and medical genetics.

From its inception to present, the Society has always considered education at the core of its remit. Within its statutes, the purpose of the Society is stated to ‘strive for the integration of scientific research and its implementation in the clinical field as well as for (postgraduate) education of specialists and the public in all areas of medical and human genetics’.^2^ As demonstration of this commitment, the Society formed ‘the Education Committee (EduComm) to disseminate the knowledge, training and teaching of modern human genetics and genomics to the general public, students, postgraduate scientists and to genetic and medical professionals’. The current, recently expanded, core membership of EduComm consists of 13 members (eight women and five men) working in 10 countries across Europe.

EduComm challenges itself to oversee a portfolio of the highest quality education and training in genomics. This has primarily been aimed at the members of the ESHG. However, there is a recognition of an additional responsibility to provide resources to health professionals and scientists working outside of genomics. Engagement and educational interaction with members of the public is also within the remit of the group. However, until now, this has not been the primary focus and we will consider how this can be addressed in more detail later. EduComm is committed to provide a broad range of educational resources. The education portfolio has targeted all levels of expertise, from a basic level understanding of genomics (where there are the greatest needs) to high-level expert training often delivered through the state-of-the-art educational symposia at the annual conference.

EduComm has sought to provide oversight and guidance for a portfolio of training courses that meet the needs of the membership (Table 1). The Society has placed a high emphasis on ensuring that these courses are affordable, aiming to ensure equitable access and the highest educational quality. Through the EuroGEMS^3^ website (Tobias and Tobias, 2020), EduComm has facilitated access by multiple audience types to a wide range of the existing high-quality, free and online educational resources in genetics and genomics, including apps and massive open online courses (MOOCs). The Committee also provides input into the ESHG Scientific Program Committee to suggest educational content for the annual conference.

**Table 1:** Current educational courses co-organised and/or supported by ESHG (https://www.eshg.org/courses.0.html ).

Course	Location	Co-organisers
Introduction to the statistical analysis of genome-wide association studies	London, United Kingdom	University of Surrey
Clinical Genomics and NGS Course	Bertinoro, Italy	University Residential Center of Bertinoro
Eye Genetics Course	Bertinoro, Italy	University Residential Center of Bertinoro
Basic and Advanced Course on Genetic Counselling	Bertinoro, Italy	University Residential Center of Bertinoro
Course in Hereditary Cancer Genetics	Bertinoro, Italy	University Residential Center of Bertinoro
Cardiac Genetics Training Course	Manchester, United Kingdom Antwerp, Belgium	Manchester Centre for Genomic Medicine
		University of Antwerp
Basics in Human Genetic Diagnostics – A course for Clinical Laboratory Geneticists in education	Various	University of Jena
Manchester Dysmorphology Course	Manchester, United Kingdom	Manchester Centre for Genomic Medicine
Goldrain Course on Clinical Cytogenetics	South Tyrol, Italy	
Pharmacogenetics	Slovenia	University of Ljubljana
Next-generation Reproductive Genetics	Netherlands	Maastricht University Medical Center

The analysis of genomic information forms an increasingly important component in the diagnosis, treatment and prevention of disease. At present, genomic medicine is only an integral part of service provision in a few countries, yet this adoption is set to expand. The ESHG has a role to support this expansion. Better use of technology and data is a prerequisite for supporting and enabling key educational developments. The COVID-19 healthcare crisis accelerated the transition to digitalisation by the rapid establishment of virtual conferences and meetings and a widespread move to online teaching. For successful integration, it is important that educators make the most of the opportunities afforded by digital education, including apps and MOOCs.

## Challenges to Delivering Genomic Education

The ESHG is just one of many organisations operating to provide educational support to health professionals and to engage with the public and patients. It is important that it targets its resources for maximal impact.

Genetic professionals face the challenge of finding a balance between their own continuous professional development and mainstreaming genomic education for other specialties. Most individuals who have a genomic test as part of their diagnostic process will, in future, have this requested, and the results managed, by non-genetic specialists. The relative lack of education in genomics for non-specialists is a challenge for implementation. Ensuring access to this expertise, training and education is complex. Many approaches with a major educational component have been adopted and led by genetic specialists, including online networks like Dyscerne (Douzgou et al., 2016b), expert resources for rare conditions, such as Orphanet (Aymé, 2003) and the educational programmes of the European Reference Networks (ERN; Tumiene and Graessner, 2021).

The ESHG is keen to understand and address the disparities in the access to training and education of health professionals in some nations and across ethnic groups. EduComm is aware of and concerned about these disparities and is defining strategies to reduce these, by facilitating access to genetic information in different languages and exploring strategies to better integrate minorities in ESHG activities. The roles of different professionals in genomics, including genetic counsellors, can be promoted through education, explaining the key skills of this workforce which has not been equally represented or formally recognised in all European countries.

The increasing digitalisation of educational resources has facilitated their global outreach but has also modified the learning experience. Traditional healthcare education and development included experiential learning and contact with the patient (for example, ward rounds), aspects less feasible in digitalised environments as they include sharing patient-sensitive data. There is an urgent need to ensure that these resources are available in different languages, with information governance and straightforward navigation by health professionals, patients and the public. The translation of the EuroGEMS website (see below) and its inclusion of multi-language resources will increase access to such resources.

Whilst online education is a powerful adjunct, many learners experience difficulty in concentrating for extended periods. Instead, short periods of engaging interactively-delivered information are fundamental to the success of online learning. Despite its cost, face-to-face teaching and experiential work remain a vital part of training and should be supported.

Research funding is not equally distributed across countries within the EU (Lynch and Borg, 2016). This may reduce the opportunities for a highly capable trainee to undertake research within their country. Some rare diseases cluster in specific geographical areas, and local research and educational resources are imperative to improve care for that specific disorder. The overlap between research and teaching is symbiotic; centres without research will encounter greater difficulties in providing leading-edge teaching.

In future, health professionals in genomics will be more actively involved in clinical trials and in treating patients. This is true of laboratory staff also, who will need to reduce test turnaround times and analyse biomarkers that indicate response or resistance to treatment. It is imperative that educational resources support the change in professional roles and ensure that up to date accurate information is available. Examples of resources in this area are starting to emerge, like Treatable-ID,^4^ which provides easily accessible information in the form of an app on treatable causes of intellectual disability.

## Educational Courses

Given the broad background, levels of experience and knowledge of ESHG members, educational courses, organised or supported by the Society, have tried to reflect the needs of the different groups. They support some of the core competencies that provide an appropriate framework for genetics education of health professionals across national boundaries (Skirton et al., 2010). The courses seek to disseminate best standards of good clinical, laboratory and data analysis practice in genetics. The topics span basic sciences to clinical delivery of Genomic Medicine and Genetic Counselling. The courses have adopted a mixed approach, including lectures, workshops, case discussions and presentations and importantly a forum to meet and share experiences and create a relaxed interactive environment for faculty and delegates. This has been more challenging throughout the COVID-19 pandemic where some courses have moved to an exclusively online format. However, this shift will likely allow a mixed approach to these courses in future, permitting many individuals to participate in a virtual capacity, whereas others will attend in person and be able to experience the full benefit of small group work and face-to-face interaction. The online format greatly democratises accessibility to courses, where financial and several other factors can prevent travel, attendance and participation for many individuals, and it will allow more flexible, greater and longer-term use of the resources.

Recent courses have been designed to consider newer areas of genomics, including pharmacogenetics, genomic sequencing, bioinformatics, statistical genetics and new technologies in prenatal medicine. Many of the courses encourage participants from non-genomic specialties to attend; for example, the cardiac genetics course has an attendance comprising 50% cardiologists. Plans are in development to expand the course portfolio to include precision medicine and biochemical genetics. Many courses are delivered in partnership with other professional societies to increase their reach and to reduce duplication.

Dysmorphology workshops and courses have been a mainstay of the ESHG portfolio (Donnai, 2017). These have been crucial in educating professionals in a field which is integral to the care of patients and families with rare inherited developmental disorders (Douzgou et al., 2016a). The interactive forum created by the dysmorphology courses forged an environment from which the Young Geneticists’ Network and the European Society of Human Genetic–Young Committee (ESHG-Y) were founded. In this way, the representation of young geneticists in the decision-making process of the ESHG was achieved and highlighted their specific educational and training requirements.

The five-day course ‘Introduction to the statistical analysis of genome-wide association studies (GWAS^5^)’ has been running yearly since 2016. The course covers basic statistical theory in GWAS, quality control, imputation, population stratification, trans-ethnic GWAS and principles of Mendelian Randomisation. Due to Covid-19, the course moved to an online live format, whilst maintaining the same content and expanding the audience. The digital platform enabled hands-on computer exercises with supervision and feedback in real time. The course, initially targeting postdoctoral researchers, has seen ~60% PhD student audience, contributing to advanced training of early career researchers in Europe and other countries worldwide.

## EuroGEMS

### Purpose and Content

To provide high-quality educational resources for genetics and (increasingly-importantly) non-genetics health professionals and the general public, an educational website at www.EuroGEMS.org has been established on behalf of EduComm (Tobias and Tobias, 2020). With high-speed, high-bandwidth, secure-socket-layer hosting, it provides content summaries and direct links for a wide range of carefully selected international online educational genetic and genomic resources, for audiences of all levels, including the public.

General inclusion criteria used in resource selection are as follows: (a) free-to-access, (b) informative, (c) containing up-to-date unbroken links, and (d) of high quality and free of obviously misleading information. Resources were chosen to include those used from personal experience and following discussions with many professional colleagues, including EduComm and the ESHG Board. Links to >110 educational resources (many from outside Europe) were thus included, categorised in web pages according to target audience. A brief summary of content and purpose was provided for each resource. The website’s content has undergone recent detailed peer review (Tobias and Tobias, 2020).

### Design to Include Non-genetics Specialists

For non-genetics health professionals, such as primary-care physicians and non-genetics (‘mainstreaming’) specialists, a web page was added with information and links, including Gen-Equip, Orphanet, GeneReviews, Unique and Contact, a range of free genomic MOOCs and several smartphone apps (described below).

### Genomic Education for the Public

A large part of EuroGEMS provides public education, including pages for patients and their relatives. These pages contain, for example, well-established online directories of rare conditions and support organisations, such as Unique, Contact, Orphanet, EURORDIS and MedlinePlus-Genetics, with more general genetics educational resources, including a range of animated videos. Other pages comprise multiple resources for primary and secondary/high school teachers, including animations and professionally filmed videos of children relating experiences of rare disorders. Pages and links of general interest are as follows: a web page of Ethical, Legal & Social Implications (ELSI) resources; links provided within several pages to many non-English and multi-language resources; access to sets of genetics-relevant COVID-19-related educational resources; and links to several MOOCs.

### International Use

EuroGEMS has received visits from 120 countries, with 47% of web page visits originating in European countries with a non-English primary language and 21% from outside Europe (Table 2). The frequency of returning visits has markedly increased, now three-fold greater (in January–March, 2021) than in the first 3months. Although the majority (55.2%) of the website’s visitors have accessed it directly *via* its URL, a large proportion (23.7%) reached it *via* the ESHG website. The users categorised by total page views are as follows: genetics professionals: 23.5%, students: 22.2%, secondary schools: 16.2%, patients and families: 13.9%, non-genetics health professionals: 10.3%, primary schools: 8.4% and ELSI: 5.4%.

**Table 2:** The 10 countries/areas inside and 10 outside the continent of Europe from which visits to EuroGEMS.org have most frequently originated.

Country/Area	Proportion (%) of total page views
Within the continent of Europe	
United Kingdom	29.8
Italy	5.2
Belgium	3.8
France	3.4
Spain	3.2
Sweden	3.2
Portugal	3.1
Germany	3.0
Turkey	2.9
Netherlands	2.5
Outside Europe	
United States	5.2
Canada	1.9
Australia	1.6
India	1.4
Brazil	0.8
Israel	0.7
China	0.7
Saudi Arabia	0.7
Egypt	0.6
Japan	0.6

*Data: Statcounter (Accessed March 16, 2021).*

### Evaluation

Highly appreciative feedback regarding EuroGEMS was received from clinical professionals, teachers and the public, internationally (including from Australia, South Africa and Saudi Arabia). Comments have included ‘really easy to use and well organised’; ‘very useful’; and ‘fantastic and I look forward to using it for the rest of my career’.

### Further Development of EuroGEMS

Since the website’s launch, its numerous links have been regularly updated and further international resources and a page for non-genetics health professionals have been added. Supported by the ESHG, translation of EuroGEMS into non-English languages is underway, commencing with Spanish.

### Massive Open Online Courses

Links within EuroGEMS to a range of MOOCs provide worldwide access to these free public-oriented extensive, cutting-edge courses, covering genetics, genomic medicine, cancer genomics, clinical bioinformatics and sequencing technologies. Designed for non-genetics healthcare professionals and the public, the MOOCs are provided on dedicated interactive online platforms and contain many short video lectures and articles, plus web links, glossaries, self-assessment quizzes and educator-moderated discussion forums. The enormous global access to the courses provided by such online delivery has, for example, enabled the genomic medicine MOOC^6^ (created by author EST and Glasgow University colleagues), to educate 805 learners (including the public, hospital physicians, primary-care practitioners, students and scientists) in 97 countries, in the first 3months post-launch.

### Smartphone Apps

The smartphone and tablet apps linked from EuroGEMS include a (Guy’s Hospital) cancer genetics referral guide for general practitioners, the Treatable-ID app (mentioned above) and a set of five free educational apps designed and coded by an EduComm member (Tobias and Tobias, 2015). This set comprises an illustrated glossary for genomics and bioinformatics terminology, greatly expanded since launch, and also self-assessment quiz apps for terminology and genetic inheritance mechanisms. Downloaded free-of-charge from Apple and Android app stores these have been used by >5,000 individuals in >70 countries (05/04/2021).

## Annual Conference

Each year the Society convenes a conference in different cities across Europe. The focus each year is to consider research at the forefront of human genetics. Equally important and integral to the conference programme is an educational track, which provides educational symposia and workshops across a broad range of topics. The EduComm has added to the programme half-day courses on specific topics with a focus on important basic standards, including accurate description of variants, describing phenotypes (Human Phenotype Ontology), variant classification, databases and data sharing. These initiatives will complement other courses provided by collaborating organisations, for example, the HVP/HUGO Variant Effect Prediction Training Course.^7^ The conference also includes a wide-ranging ELSI-related multi-session track entitled Ethical Legal and Psychosocial Aspects in Genetics led by the ESHG Public and Professional Policy Committee.

## Mentorship

The EduComm established a mentorship scheme to broaden the opportunity for young ESHG members to benefit from interaction with another centre. The mentorship scheme will especially support individuals from economically disadvantaged countries or where genetics services are less well developed. The successful candidates will combine attendance at the annual conference with a visit to the centre of an established international leader in any field of genomics or relevant subject area. We hope that this scheme will act as an adjunct to the ERN clinical exchange programmes and foster long-term supportive relationships between the mentor and the young Society member, enhancing their career progression.

## Public and Patient Education in Genomics

The ESHG has previously had a more limited role in educating the wider non-scientific public in genomic and precision medicine. Interaction with school children has previously been at the centre of the approach to public engagement taken by the ESHG. Currently, events are hosted for school children aligned to the annual conference.

Many ESHG members work with patient advocacy groups providing expert scientific and medical advice. Through this, the accurate information can be shared with affected individuals, their families and carers for a broad range of rare conditions where there is less knowledge within the healthcare system. Additionally, ESHG-Y members are supporting the translation of the Unique^8^ patient guides into seven languages. Social media can act as a form of knowledge and information exchange and has been adopted by many patient organisations. Several organisations have established websites and are leading condition-specific, educational strategies, e.g., for Myhre syndrome.^9^

## The DNA Day Essay Contest

DNA Day, April 25, is commemorated internationally as a celebration of genetics. The ESHG sponsors a DNA Day Essay contest in European high schools. The contest is designed as a learning tool and a means to promote knowledge of genetics within Europe. It intends to challenge students to examine, question and reflect on the importance and social implications of genetic research and its applications. Essays are expected to contain substantive, well-reasoned arguments indicative of a depth of understanding of the issues. The essays received have been of excellent quality with award winners from throughout Europe.

## Conclusion

The challenges and opportunities to meet the needs of its members, health professionals, scientists, patients and the public, with the increasing adoption of genomics in healthcare, are enormous. The ESHG is just one organisation contributing to education and training in genomics. Work with the educational committees of other international genomic societies (e.g. the American Society of Human Genetics), universities, hospitals, patient support groups and commercial partners to deliver and support educational activities will be important to maximise the impact of genomic and precision medicine. Taking flexible, complementary approaches to the education and training of individuals, irrespective of their experience, role and location, will be key to the success of creating a genomics-literate society.

## Data Availability Statement

The original contributions presented in the study are included in the article/supplementary material; further inquiries can be directed to the corresponding author.

## Author Contributions

All authors listed have made a substantial, direct and intellectual contribution to the work, and approved it for publication.

## Conflict of Interest

PP was employed by company NIPD Genetics Limited, Cyprus and CC was employed by company CC-SYNLAB Suisse SA, Switzerland.

The remaining authors declare that the research was conducted in the absence of any commercial or financial relationships that could be construed as a potential conflict of interest.

## Publisher’s Note

All claims expressed in this article are solely those of the authors and do not necessarily represent those of their affiliated organizations, or those of the publisher, the editors and the reviewers. Any product that may be evaluated in this article, or claim that may be made by its manufacturer, is not guaranteed or endorsed by the publisher.

## Footnotes

^1^ https://www.eshg.org/

^2^ https://www.eshg.org/index.php?id=54

^3^ https://www.eurogems.org

^4^ https://www.treatable-id.org/

^5^ https://www.eshg.org/index.php?id=104

^6^ https://www.futurelearn.com/courses/harnessing-the-power-of-genomics-in-medicine

^7^ VEPTC.variome.org

^8^ https://rarechromo.org/

^9^ https://www.myhresyndrome.org

## References

[ref1] Aymé S. (2003). Orphanet, un serveur d’informations Sur les maladies rares [Orphanet, an information site on rare diseases]. Soins 672, 46–47. PMID: 12655825

[ref2] Donnai D. (2017). Dysmorphology and the ESHG. Eur. J. Hum. Genet. 25, S33–S34. 10.1038/ejhg.2017.161, PMID: 29297884 PMC5763268

[ref3] Douzgou S. Chervinsky E. Gyftodimou Y. Kitsiou-Tzeli S. Shalev S. Kanavakis E.. (2016a). Dysmorphology services: a snapshot of current practices and a vision for the future. Clin. Genet. 89, 27–33. 10.1111/cge.12571, PMID: 25683496

[ref4] Douzgou S. Pollalis Y. A. Vozikis A. Patrinos G. P. Clayton-Smith J. (2016b). Collaborative crowdsourcing for the diagnosis of rare genetic syndromes: the DYSCERNE experience. Public Health Genomics 19, 19–24. 10.1159/000440710, PMID: 26447648

[ref5] Lynch S. A. Borg I. (2016). Wide disparity of clinical genetics services and EU rare disease research funding across Europe. J. Community Genet. 7, 119–126. 10.1007/s12687-015-0256-y, PMID: 26536881 PMC4796048

[ref6] Renwick J. H. Edwards A. W. (1995). The foundation of the european society of human genetics. Eur. J. Hum. Genet. 3, 63–64. PMID: 7552143

[ref7] Skirton H. Lewis C. Kent A. Coviello D. A. Members of Eurogentest Unit 6 and ESHG Education Committee (2010). Genetic education and the challenge of genomic medicine: development of core competences to support preparation of health professionals in Europe. Eur. J. Hum. Genet. 18, 972–977. 10.1038/ejhg.2010.64, PMID: 20442748 PMC2987423

[ref8] Tobias A. P. Tobias E. S. (2015). Developing educational iPhone, android and windows smartphone cross-platform apps to facilitate understanding of clinical genomics terminology. Appl. Transl. Genom. 28, 15–17. 10.1016/j.atg.2015.08.001, PMID: 27054073 PMC4803764

[ref9] Tobias A. P. Tobias E. S. (2020). EuroGEMS.org: guide and links to online genetic and genomic educational resources, valuable for all levels. Hum. Mutat. 41, 2021–2027. 10.1002/humu.24113, PMID: 32906220

[ref10] Tumiene B. Graessner H. (2021). Rare disease care pathways in the EU: from odysseys and labyrinths towards highways. J. Community Genet. 12, 231–239. 10.1007/s12687-021-00520-9, PMID: 33738760 PMC8141079

